# Factors Associated with Exercise Adherence Across Settings Among Stroke Survivors in China: A Systematic Review of Cross-Sectional Studies

**DOI:** 10.3390/healthcare14162475

**Published:** 2026-08-10

**Authors:** Su Sean Yong, Qifei Chen, Zhiping Liao, Fangchao Wu, Jianhua Li, Qi Si

**Affiliations:** 1 Department of Sports Science, College of Education, Zhejiang University, Hangzhou 310058, China; suseanyong@zju.edu.cn (S.S.Y.); 22303045@zju.edu.cn (Q.C.); 2 Department of Physical Medicine and Rehabilitation, Sir Run Run Shaw Hospital, School of Medicine, Zhejiang University, Hangzhou 310016, China; 3316068@zju.edu.cn (Z.L.); 3414030@zju.edu.cn (F.W.)

**Keywords:** factors, exercise adherence, in-hospital, post-discharge, stroke survivors

## Abstract

**Background/Objectives:** Exercise is an important component of rehabilitation programmes for stroke survivors, and adherence is key to ensuring the effectiveness of rehabilitation. However, adherence declines over time, especially as stroke survivors transition from in-hospital to post-discharge settings. The present review synthesised evidence on factors associated with exercise adherence among stroke survivors in China across two settings, using the socio-ecological model. This could provide a basis for developing effective intervention strategies to improve exercise adherence. **Methods:** A literature search was conducted via China National Knowledge Infrastructure, Wanfang, Weipu, PubMed, and Web of Science from inception to 30 December 2025. Only cross-sectional studies that examined factors associated with exercise adherence were included. Studies were screened against the inclusion and exclusion criteria. Data were extracted, categorised, and analysed using a semi-quantitative approach. The review protocol was not prospectively registered but was later registered on the Open Science Framework. **Results:** Twenty-three studies were included, of which 13 focused on the in-hospital setting and 10 on the post-discharge setting, published between 2013 and 2025, involving 7557 stroke survivors aged 18 to over 75 years, with a mean quality assessment score of 85.33%. Exercise adherence was generally at a medium level in both settings, with a slightly lower adherence index after discharge. During hospitalisation, 34 intrapersonal and five interpersonal factors were identified, while 29 intrapersonal, five interpersonal, and one environmental factor were identified after discharge. Sufficient evidence showed that educational level, functional independence, and self-efficacy were positively associated with exercise adherence across both settings. During hospitalisation, social support was also positively associated with adherence, while age and stroke severity were negatively associated. After discharge, marital status, knowledge, and attitude were positively associated, while time since stroke onset and balance impairment were negatively associated. **Conclusions:** Previous studies mainly focused on intrapersonal factors, followed by interpersonal factors, while environmental factors received limited attention. Future studies should examine potentially modifiable factors at all three levels and determine whether addressing these factors supports exercise adherence among stroke survivors during hospitalisation and after discharge.

## 1. Introduction

Stroke is the second leading cause of death and the third leading cause of disability worldwide, with approximately 7 million deaths and over 160 million disability-adjusted life years lost in 2021 [1]. In China, the burden is particularly considerable, with about one-third of global stroke deaths each year [2], and stroke is now the leading cause of death and disability [3]. According to the Global Burden of Disease Study 2019, the number of deaths due to stroke reached 2.19 million, while the number of disability-adjusted life years lost due to stroke reached 45.9 million [3]. Both measures increased substantially between 1990 and 2019 and are projected to continue to increase over the next three decades [4,5]. Thus, stroke is a major public health problem in China.

Stroke can lead to impairments in physical, cognitive, psychological, and/or social functioning [6]. These impairments can limit functional capacity, hinder the ability to perform activities of daily living, and affect quality of life. Exercise has been shown to mitigate these effects and decrease the risk of subsequent cardiovascular events [7]. Exercise is an important component of stroke rehabilitation, and adherence to exercise is essential to maximise its effectiveness [7]. The World Health Organization defines adherence as “the extent to which a person’s behaviour—taking medication, following a diet, and/or executing lifestyle changes, corresponds with agreed recommendations from a health care provider” [8]. In the context of stroke rehabilitation, however, exercise adherence is a complex and multidimensional construct [9,10,11], involving individuals taking an active role in treatment [12]. 

Adherence to exercise among stroke survivors also changes over time. A longitudinal study found that adherence increased rapidly during the first 6 weeks of hospitalisation, declined slowly from weeks 6 to 21 as most survivors were discharged home, and remained stable from weeks 21 to 24 [13]. This pattern reflects the transition from in-hospital to post-discharge settings, indicating that factors associated with adherence might differ between settings. Health care services and pathways, as well as cultural context in China, might also differ from other countries, which could limit the applicability of international findings to stroke survivors in China. However, a previous review of factors associated with exercise adherence among stroke survivors used a narrative synthesis that mainly provided a descriptive summary, without comparing factors across settings, and was not specific to China [14]. A synthesis of evidence that is specific to China across settings is therefore needed. 

The socio-ecological model is suitable for this purpose as it conceptualises health behaviour broadly and emphasises interactions among factors at multiple levels rather than focusing solely on individual factors [15]. This is particularly relevant to stroke rehabilitation, as exercise adherence does not occur in isolation but within wider contexts. Summarising factors according to the socio-ecological model could provide a more comprehensive understanding of exercise adherence. The present review focused on cross-sectional studies, aiming to synthesise factors associated with adherence to exercise among stroke survivors in China, compare these factors across settings, identify those common to both settings, and classify them according to a three-level socio-ecological model (i.e., intrapersonal, interpersonal, and environmental) [16]. The review also used a semi-quantitative approach to summarise the direction and consistency of associations. Overall, the findings might provide a basis for future research and the development of strategies to support exercise adherence. 

## 2. Methods

The systematic review was conducted and reported according to the Preferred Reporting Items for Systematic Reviews and Meta-Analyses (PRISMA) guidelines. This review was retrospectively registered with the Open Science Framework under the registration DOI: 10.17605/OSF.IO/432PQ and is available at https://osf.io/432pq/ (accessed on 7 July 2026).

### 2.1. Search Strategy

A literature search was conducted in Chinese and English databases, including the China National Knowledge Infrastructure (CNKI), Wanfang, Weipu, PubMed, and Web of Science. As this review focused on stroke survivors in China, the three major Chinese databases were searched for local studies, while two broad English databases were searched to identify relevant studies published in English. The search was restricted from the inception of each database to 30 December 2025. Four key search concepts were built around population (e.g., stroke, cerebrovascular accident), exercise (e.g., exercise, physical activity), outcome (e.g., adherence, compliance), and relevant factors (e.g., determinants, correlates). The reference lists of the included studies were also searched manually to identify any additional relevant studies. There was no language restriction for the search. Details of search terms are presented in the Supplementary File S1.

### 2.2. Eligibility Criteria

In the present review, exercise adherence was operationally defined as the extent to which stroke survivors follow agreed exercise recommendations across relevant exercise parameters, such as frequency, intensity, and duration, and actively manage and continue their exercise as recommended.

Studies were included if they: (i) involved stroke survivors who were ≥18 years of age; (ii) measured exercise adherence and explored its associated factors; (iii) conducted multivariable analyses to reduce potential confounding bias; (iv) were cross-sectional studies; and (v) were published in Chinese or English. Studies were excluded if they: (i) did not clearly report the setting (i.e., not stated whether data collected during hospitalisation or after discharge) and still could not be classified into either setting after attempts to contact the corresponding author, as no response was received; (ii) did not clearly report how exercise adherence was measured or how adherence levels were classified; (iii) measured treatment adherence but exercise-only data could not be obtained; (iv) conducted only univariate and correlation analysis; or (v) were reviews, meta-analyses, controlled trials, qualitative studies, cohort studies, case reports, dissertations, or conference abstracts.

### 2.3. Selection Process

After duplicates were removed, titles and abstracts were screened by two independent reviewers (S.S.Y. and Q.C.) to exclude articles that were not relevant. The full texts of relevant articles were sought and assessed for eligibility. Any disagreements were resolved by discussion between the two reviewers, and if necessary, the third reviewer (Q.S.) was consulted.

### 2.4. Quality Assessment

The methodological quality of the included studies was independently evaluated by two reviewers (F.W. and Z.L.) using the 8-item Joanna Briggs Institute (JBI) Critical Appraisal Checklist for analytical cross-sectional studies. Each checklist item was scored as follows: “Yes” = 1, “No” = 0, and “Unclear” = 0.5. The scoring and percentage thresholds were adapted from a previous review [17]. An “Unclear” response was assigned 0.5 to distinguish incomplete reporting from a criterion that was clearly not met. The total quality score was calculated using the following formula: Total score = (sum of item scores/8) × 100%. Studies were categorised as good (scores ≥ 70%), acceptable (scores 50–69%), and low (scores < 50%). The item-level appraisal was also considered, as the total score might not reflect all methodological concerns. Any disagreements between the two reviewers were first resolved through discussion. A third reviewer (J.L.) was consulted if consensus could not be reached.

### 2.5. Data Extraction

All data were extracted by two independent reviewers (S.S.Y. and Q.C.) and were cross-checked. Extracted information included first author, year of publication, sample size, age, setting, measurement tool, adherence outcomes (i.e., adherence index and distribution of adherence level), and associated factors. Possible overlap between study samples was checked, and results for the same factors from potentially overlapping samples were counted only once. Studies conducted during hospitalisation were categorised as “in-hospital”, while those conducted in outpatient, community, or home-based settings were categorised as “post-discharge”. Studies that reported only a total score of adherence were converted to an adherence index using the formula: Adherence Index = (total score/maximum score) × 100 [18]. If the total score was not provided and only gender-specific means and standard deviations were available, StatsToDo (https://www.statstodo.com/CombineMeansSDs.php (accessed on 12 February 2026)) was used to combine the data, which was then converted into an adherence index. 

### 2.6. Data Synthesis

When the adherence index was reported, the overall adherence level was reclassified as high (≥75%), medium (50–74.9%), and low (<50%) according to the cut-offs proposed by Lin and colleagues [18]. If the adherence index could not be obtained and only the adherence level distribution was reported, the overall adherence level was determined as the category with the largest proportion of participants (>50%). When no category accounted for at least 50% of participants, the overall adherence level was reclassified as a mixed category combining two adjacent categories with the largest proportions, and described as low-to-medium or medium-to-high.

Factors associated with exercise adherence were categorised as “positive association” or “negative association” when the association was statistically significant (*p* < 0.05), otherwise, they were considered as “no association”. Only adjusted findings from multivariable analyses were included, and associations were counted according to the reported direction and statistical significance of the adjusted results. Studies using different exercise adherence measurement tools were synthesised when they met the eligibility criteria of the review. Variables that used different terms or measures were grouped under a broader factor when they represented closely related concepts. Variables that clearly represented different concepts were kept separate. The identified factors were then classified according to the socio-ecological model as intrapersonal, interpersonal, and environmental. The factors were also grouped by study setting (i.e., in-hospital and post-discharge).

### 2.7. Semi-Quantitative Synthesis

A semi-quantitative approach was then applied to summarise the consistency of evidence. Following the method used in a previous study conducted by Sallis and colleagues [19], two parameters were calculated for each factor: (1) the number of studies showing a statistically significant association between exercise adherence and the factor, and (2) the total number of studies examining that association. The proportion of studies supporting the association was calculated as: (Parameter 1/Parameter 2) × 100%. If the proportion was 0–33%, the association was coded as “0”, indicating no association. If the proportion was 34–59%, the association was coded as “?”, indicating inconsistent findings. If the proportion was 60–100%, the association was coded as “+” or “−”, indicating a positive or negative association, respectively. The amount of evidence was considered limited when the factor was examined in fewer than three studies, regardless of the consistency of the findings. The evidence was considered sufficient when three or more studies were available and met the consistency threshold. In such cases, the original codes were doubled as “00”, “++”, “−−”, or “??”, indicating sufficient evidence of no association, positive association, negative association, or inconsistent findings, respectively. NA was used when a factor was reported by only one study, as further synthesis was not possible.

## 3. Results

### 3.1. Study Selection

**Figure 1:**
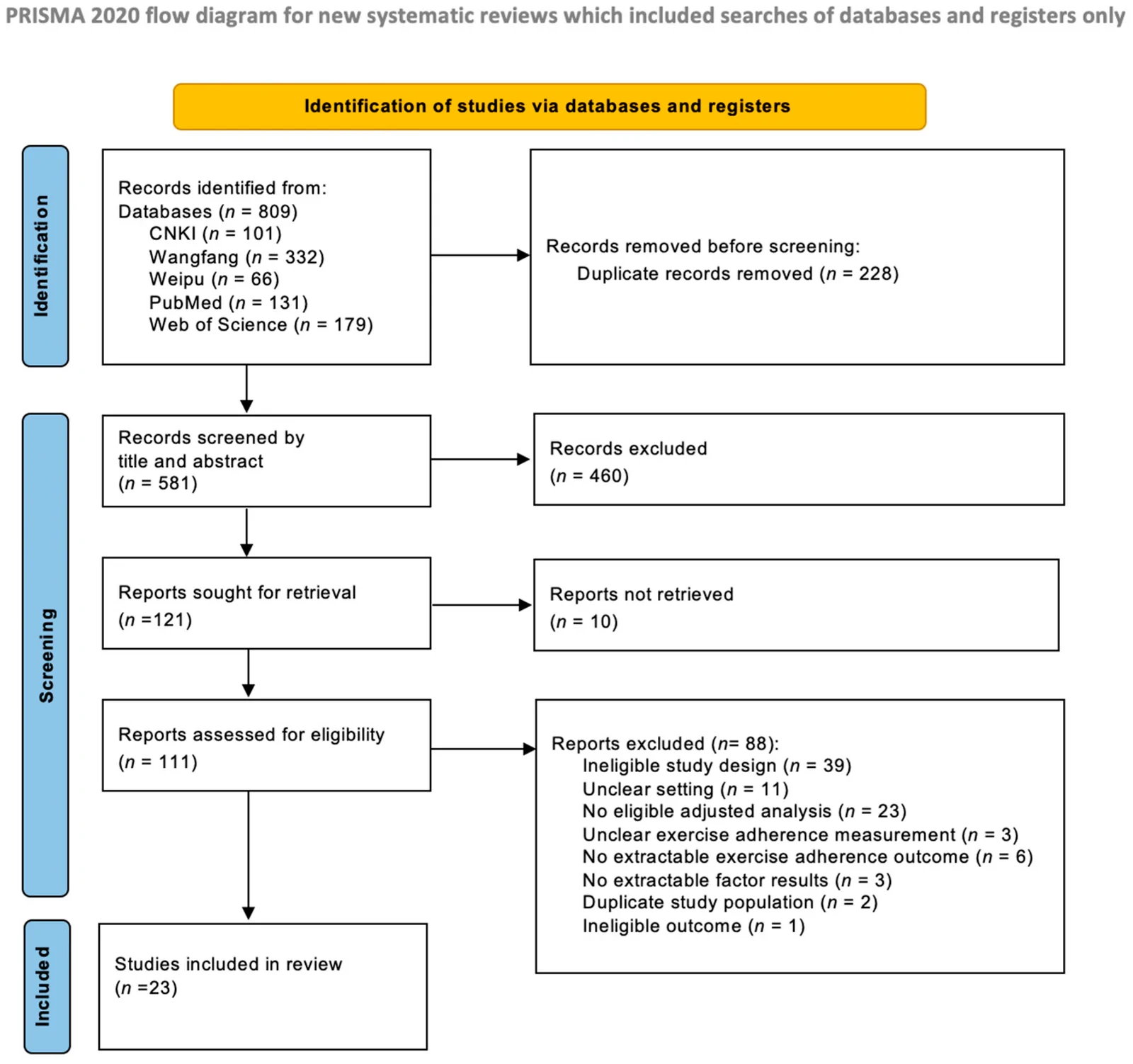
PRISMA Flowchart of the study selection process.

A total of 809 studies were identified through database searches. After removing 228 duplicates, 581 titles and abstracts were screened, and 121 potentially relevant studies were sought for retrieval, of which 10 could not be retrieved. The remaining 111 studies were assessed for eligibility, and 23 studies met the inclusion criteria and were included in the present review. The study selection process is shown in Figure 1.

### 3.2. Methodological Quality

The methodological quality scores ranged from 68.75% to 93.75%, with a mean score of 85.33% and a median score of 87.50%. Most of the studies (95.65%) were rated as good quality, while one study (4.35%) was rated as acceptable quality. Details of the methodological quality assessment are presented in Table 1.

### 3.3. Study Characteristics

The main characteristics of included studies, including author, year, sample size, age, setting, and measurement tool, are summarised in Table 2. All of these studies became available between 2013 and 2025. Nine studies (39.10%) were conducted in eastern China, four (17.40%) in southwestern China, three (13.00%) in central China, two (8.70%) each in northern and southern China, one (4.30%) in northwestern China, and another two (8.70%) were conducted across multiple regions of China. The included studies comprised a total of 7557 stroke survivors aged 18 to over 75 years. Thirteen studies focused on the in-hospital setting, while the remaining ten studies focused on the post-discharge setting. Twenty studies used the Exercise Adherence Questionnaire (EAQ). One study used the Compliance Scale of Functional Exercise (CSFE), and two studies used self-developed questionnaires. 

Exercise adherence outcomes, including adherence index, adherence level distribution, and overall adherence level, are summarised in Table 3. The adherence index for stroke survivors during hospitalisation ranged from 54.83% to 77.71%, and from 53.89% to 66.19% after discharge. Four studies did not report the adherence index and only provided the distribution of adherence levels. In the in-hospital setting, the overall adherence level was classified as high in 2 studies, medium in 10 studies, and low in 1 study. In the post-discharge setting, it was classified as medium-to-high in 2 studies and medium in 8 studies.

### 3.4. Associations Between Factors and Exercise Adherence Across Settings

A total of 39 factors associated with exercise adherence among stroke survivors during hospitalisation were identified, of which 34 were intrapersonal factors and 5 were interpersonal factors (Table 4). There was sufficient evidence for positive associations between exercise adherence and educational level (5/8, 63%), functional independence (5/6, 83%), self-efficacy (3/3, 100%), and social support (3/3, 100%), whereas age (4/4, 100%) and stroke severity (3/3, 100%) were negatively associated with exercise adherence. Limited evidence suggested positive associations with marital status (2/3, 67%) and family functioning (2/2, 100%), and a negative association with psychological distress (2/3, 67%). Evidence on gender, average income, payment method, muscle strength, and communication impairment was inconsistent.

**Figure 2:**
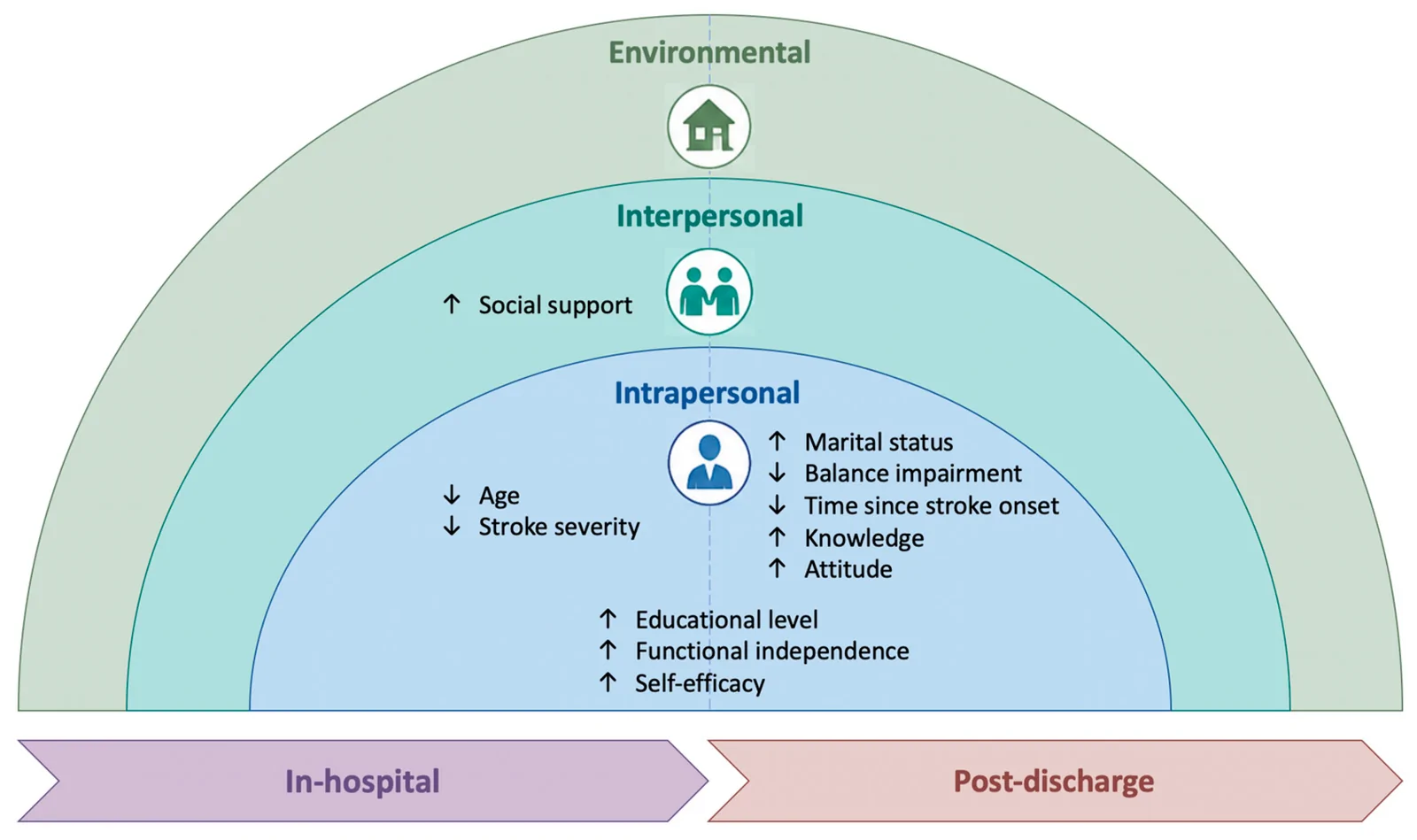
Summary of factors associated with exercise adherence across settings within the socio-ecological model. “↑” indicates a positive association; “↓” indicates a negative association; only factors supported by sufficient evidence are shown; environmental factors remained under-examined; factors with limited or inconsistent evidence are reported in Table 4 and Table 5.

After discharge, 32 factors associated with exercise adherence among stroke survivors were identified, including 26 intrapersonal factors, 5 interpersonal factors, and 1 environmental factor (Table 5). The evidence was sufficient to support positive associations between exercise adherence and marital status (3/4, 75%), educational level (7/7, 100%), functional independence (3/3, 100%), knowledge (3/3, 100%), attitude (3/3, 100%), and self-efficacy (3/3, 100%), whereas time since stroke onset (3/4, 75%) and balance impairment (3/3, 100%) were negatively associated with exercise adherence. Limited evidence suggested that family functioning (2/2, 100%) and assistive equipment (2/2, 100%) were positively associated with exercise adherence, whereas stroke severity (2/2, 100%), hemiplegia (2/2, 100%), communication impairment (2/2, 100%), and psychological distress (2/2, 100%) were negatively associated. Evidence on age and average income was inconsistent. A summary of factors associated with exercise adherence across both settings within the socio-ecological model is illustrated in Figure 2.

**Table 5:** Summary of factors associated with exercise adherence (post-discharge).

Variables		Positive Association, *n*	Negative Association, *n*	No Association, *n*	*n*/*N* (%)	Strength of Evidence
**Intrapersonal**	**Demographic factors**					
	Age		2	2	2/4; 50%	?
	Gender (female)			18	0/1; 0%	NA
	Marital status (married)	3		1	3/4; 75%	++
	Place of residence			1	0/1; 0%	NA
	Educational level	7			7/7; 100%	++
	Average income	1		1	1/2; 50%	?
	Employment status (employed)	1			1/1; 100%	NA
	Payment method (health insurance)			1	0/1; 0%	NA
	**Health and clinical status factors**					
	Stroke severity		2		2/2; 100%	−
	Stroke type (haemorrhagic)			1	0/1; 0%	NA
	Time since stroke onset	1	3		3/4; 75%	−−
	Functional independence	3			3/3; 100%	++
	Physical ability	1			1/1; 100%	NA
	Number of comorbidities			1	0/1; 0%	NA
	Number of complications			1	0/1; 0%	NA
	Hemiplegia		2		2/2; 100%	−
	Dysphagia			1	0/1; 0%	NA
	Balance impairment		3		3/3; 100%	−−
	Sensory impairment			1	0/1; 0%	NA
	Communication impairment		2		2/2; 100%	−
	**Psychosocial and behavioural factors**					
	Knowledge	3			3/3; 100%	++
	Attitude	3			3/3; 100%	++
	Self-efficacy	3			3/3; 100%	++
	Health beliefs	1			1/1; 100%	NA
	Psychological distress		2		2/2; 100%	−
	Exercise habits	1			1/1; 100%	NA
**Interpersonal**	Social support	1			1/1; 100%	NA
	Family functioning	2			2/2; 100%	+
	Caregiver (child/spouse)	1			1/1; 100%	NA
	Caregiver (paid helper)			1	1/1; 100%	NA
	Rehabilitation guidance/support			1	0/1; 0%	NA
**Environmental**	Assistive equipment	2			2/2; 100%	+

Note. “*n*” indicates the number of studies reporting an association; “*n*/*N*” indicates the number and proportion of relevant studies reporting an association in the same direction out of the total studies; “+” indicates a positive association, “−” indicates a negative association, “0” indicates no association, and “?” indicates inconsistent findings; “++”, “−−” indicate sufficient consistency of evidence; NA indicates that further analysis was not possible.

## 4. Discussion

This review provided a comprehensive overview of factors at multiple levels associated with exercise adherence across in-hospital and post-discharge settings among stroke survivors in China. Exercise adherence was slightly lower after discharge. During hospitalisation, the main associated factors included social support, age, and stroke severity, while marital status, knowledge, attitude, time since stroke onset, and balance impairment were identified after discharge. Educational level, functional independence, and self-efficacy were consistently associated with exercise adherence in both settings. Most studies focused on intrapersonal factors, whereas environmental factors remained under-examined. These findings suggested that exercise adherence might require different support for in-hospital and post-discharge settings, while continued attention is also needed for factors that remain relevant in both settings.

### 4.1. Exercise Adherence Patterns Across Settings

Exercise adherence was generally at a medium level in both settings. However, the adherence index results showed that adherence to exercise after discharge tended to be slightly lower than during hospitalisation. Although this review included only cross-sectional studies, this pattern was broadly similar to previous longitudinal studies, suggesting a slight decline after discharge [13,43]. The process of recovery following a stroke often involves transitions across settings. Throughout this process, intrapersonal, interpersonal, and environmental factors might also change. However, the present review could not determine whether these factors change over time and how they relate to changes in exercise adherence. Future longitudinal studies should therefore measure exercise adherence and its associated factors over time to clarify their temporal relationships and inform more appropriate interventions. 

### 4.2. Focus of Previous Research

The socio-ecological model focuses not only on individual factors related to behavioural change, but also on social and environmental factors [44]. Such a model was used as a framework in the present review and revealed that studies in China focused mainly on intrapersonal factors, with relatively limited attention to interpersonal and environmental factors. This might provide an incomplete understanding of exercise adherence, particularly as stroke survivors transfer across settings and encounter changing interpersonal and environmental conditions. Future research should use theory-based frameworks to examine associated factors at different levels and how they interact. This could provide a stronger basis for developing and testing multilevel approaches to support exercise adherence.

### 4.3. Key Factors Associated with Exercise Adherence Across Settings

#### 4.3.1. Intrapersonal Factors

Even taking into account the differences between in-hospital and post-discharge settings, the positive association between self-efficacy and exercise adherence in stroke survivors remained evident. This was plausible based on self-efficacy theory, as individuals with higher self-efficacy are more likely to initiate and maintain exercise behaviour [45,46]. Similar findings have been reported in previous systematic reviews conducted in inpatient, outpatient, and home-based settings [47,48,49]. Future intervention studies should examine whether strategies to improve self-efficacy help support exercise adherence across settings.

Previous exercise experiences and habits might act as mastery experiences and strengthen perceived capability to engage in and maintain similar behaviours [45], which might partly explain the association between self-efficacy and exercise adherence. Previous reviews suggested that prior exercise experiences and habits were associated with exercise adherence [49,50,51]. However, only one study in the present review examined this factor. Future studies should examine this association and assess whether it might be partly explained by self-efficacy during the early phase. Stroke survivors with limited previous exercise might benefit from additional educational support, as individuals in the early phase after stroke reported relatively low exercise self-efficacy and limited information on how to perform exercise [52].

In the present review, knowledge was positively associated with exercise adherence after discharge. Immediate support and guidance from health care professionals are often reduced or unavailable after discharge, and lack of relevant knowledge might contribute to suboptimal adherence. Stroke survivors might not understand how exercise facilitates stroke recovery, what exercise to do, and what dose is recommended. A previous qualitative study suggested that limited knowledge of stroke recovery and the benefits of continued exercise might hinder long-term adherence, as stroke survivors believed that recovery occurred naturally and that exercise did not need to be continued long-term [53]. Attitude was positively associated with exercise adherence after discharge in the present review and might be partly explained by the role of knowledge. A better understanding of stroke recovery and the effects of exercise might help stroke survivors develop more positive attitudes towards continued exercise. However, this finding differed from a previous review, which found that attitude did not predict exercise adherence among stroke survivors discharged to home [49]. Therefore, the role of attitude after discharge remains unclear and should be further examined.

Educational level was another factor positively associated with exercise adherence across settings in the present review. This finding was partly supported by a previous systematic review, which found that higher education predicted better adherence to home-based exercise [49]. A possible explanation might be that individuals with higher educational levels have better knowledge and understanding of stroke rehabilitation or are better able to interpret and apply exercise advice according to their own situation. Previous studies also showed that educational level was associated with the acquisition of related knowledge [54,55]. Therefore, the association between knowledge and exercise adherence might be partly explained by educational level. Future studies should assess both factors early after hospital admission to determine whether knowledge is associated with exercise adherence independently of educational level. This might help health care professionals better identify stroke survivors who need additional support. As educational level itself is difficult to modify, support should focus on modifiable factors, such as knowledge and understanding. During hospitalisation, exercise plans need to be clearly explained to help stroke survivors develop basic exercise knowledge before discharge. Accessible support, such as video-guided exercise programmes, could then be provided before discharge or during follow-up sessions. A previous study showed that video-guided exercise improved 3-month exercise adherence [56]. Such a strategy might help stroke survivors maintain the prescribed exercise when direct supervision from health care professionals is reduced. 

Psychological distress was negatively associated with exercise adherence across settings, although the evidence was limited, with fewer than three studies supporting this association. However, this finding was supported by previous systematic reviews. A review of home-based exercise therapy in patients with chronic diseases found that lower depressive symptoms predicted better adherence to exercise [49]. Another systematic review of prescribed exercise programmes for older adults also reported that depression was negatively associated with exercise adherence, whereas good self-rated mental health was positively associated with adherence [47]. Psychological distress might affect exercise adherence through self-efficacy, as emotional states are one source of self-efficacy [57]. This was consistent with evidence showing a negative association between depression and self-efficacy among stroke survivors [58]. Early screening for psychological distress might help identify stroke survivors who need timely referral to psychological and support services to manage distress and maintain their perceived capability to adhere to exercise. 

Functional independence in activities of daily living was positively associated with exercise adherence in both settings, and this association was supported by sufficient evidence. Other health and clinical status factors were also associated with exercise adherence in different settings. There was sufficient evidence that stroke severity was negatively associated with exercise adherence during hospitalisation, while time since stroke onset and balance impairment were negatively associated with exercise adherence after discharge. These findings were generally consistent with a previous review suggesting that fewer health conditions and better physical abilities were associated with better adherence to exercise among older adults [59]. A previous longitudinal study also showed that increased adherence was associated with improved functional outcomes after stroke [60]. Therefore, better functional independence might be associated with better exercise adherence, whereas better exercise adherence might also be associated with improvements in functional independence. However, the included studies were all cross-sectional, which could not determine the direction of this relationship. Future longitudinal studies would be needed to further clarify this relationship.

#### 4.3.2. Interpersonal Factors

Social support was positively associated with exercise adherence during hospitalisation. This was generally supported by a previous review showing that social support was associated with exercise adherence among stroke survivors [14]. However, evidence on social support after discharge was limited in the present review, as only one included study examined this factor. Previous findings were also inconsistent. For example, a review found no association between social support and adherence to home-based exercise in stroke survivors [49], whereas other reviews indicated that social support was important for adherence to home physical therapy or home-based exercise [51,61]. Marital status was also associated with post-discharge exercise adherence in the present review, and being married was previously shown to be positively associated with perceived social support [62]. This might indirectly support the possible association between social support and post-discharge exercise adherence. Therefore, further studies would be needed to examine whether and how the availability and quality of social support are associated with post-discharge exercise adherence.

Family functioning was also positively associated with exercise adherence, but the evidence was not sufficient, with only two studies in each setting reporting this association. A previous study found that better family functioning was associated with lower psychological distress among stroke survivors, with perceived social support also playing a role in this association [63]. As both psychological distress and social support were associated with exercise adherence in the present review, these two intrapersonal and interpersonal factors might partly explain the association between family functioning and exercise adherence. Therefore, further studies would be needed to examine this association across settings and the possible roles of psychological distress and social support.

#### 4.3.3. Environmental Factors

Overall, the included studies indicated that environmental factors remained under-examined. One possible reason is that environmental factors are diverse and context-specific and might require broader assessment methods than the questionnaires commonly used in previous studies. There were only two post-discharge studies that examined assistive equipment, and both reported a positive association with exercise adherence. A previous review also identified exercise facilities as an environmental factor associated with adherence among stroke survivors [14]. The accessibility and availability of environmental resources might provide practical support and behavioural cues for continuing exercise after discharge [64]. 

Stroke survivors might encounter more barriers to exercise than the general population. Therefore, a supportive exercise environment suited to the functional needs of stroke survivors might be important for exercise adherence after discharge. Future research should examine a broader range of post-discharge environmental factors, including neighbourhood environments, community exercise facilities and resources, as well as home exercise equipment and space. The role of transportation in helping stroke survivors access community exercise facilities and resources should also be examined. Rehabilitation technology and digital health support should be studied separately to determine whether they help stroke survivors continue adhering to exercise after discharge. All of these factors should also be evaluated in terms of their design, accessibility, safety, and usability for stroke survivors with different functional limitations, as these features might support independent use and long-term adherence to exercise.

Environmental factors might also interact with intrapersonal and interpersonal factors. For example, access to exercise equipment and community facilities and resources might depend on family income, living space, and residential location. In the Chinese context, family members might also play an important role in rehabilitation decisions and daily exercise support by helping purchase equipment, providing transportation, offering encouragement, or being present during exercise. At a broader level, the length of hospital stay, access to community facilities and resources, and follow-up support might also depend on how rehabilitation services and pathways are organised within the health care system. These arrangements might differ between countries and limit the generalisability of the findings.

### 4.4. Strengths and Limitations

There were several limitations worth noting. Although both Chinese and English databases were searched, only two English databases were included. This might have resulted in some relevant English language studies indexed only in other English databases being missed. In addition, grey literature was not searched, which might have increased the risk of publication bias. Another limitation was that the review protocol was not registered before the review was conducted. Although it has now been retrospectively registered on the Open Science Framework, this does not provide the same level of transparency as prospective registration. Only cross-sectional studies were included. These studies could identify factors associated with exercise adherence but could not establish causal relationships. Most included studies were rated as having good methodological quality. However, some item-level methodological concerns were identified, and the findings should be interpreted cautiously. Data extraction was based on the judgment of the researchers, which might cause potential bias. However, this risk was reduced by having two reviewers independently extract and cross-check the data. In addition, a semi-quantitative synthesis was used instead of a meta-analysis. Therefore, the strength of the associations and pooled effect estimates could not be evaluated. Another limitation was that the present review only focused on stroke survivors in China, so the findings might not be generalisable to stroke survivors in other countries because of differences in cultural context, as well as health care services and pathways. 

The strengths of the present review included that only studies using multivariable analyses were included. This helped reduce the influence of potential confounding factors. Another strength was the use of the socio-ecological model as a guiding framework. This allowed factors associated with exercise adherence among stroke survivors in China during hospitalisation and after discharge to be synthesised across intrapersonal, interpersonal, and environmental levels. The findings might inform the development of more comprehensive interventions. 

## 5. Conclusions

Overall, the present review suggested that supporting exercise adherence among stroke survivors might require considering factors at multiple levels rather than focusing mainly on intrapersonal factors. As all included studies were observational, causal relationships could not be determined. Future intervention studies should examine whether improving potentially modifiable factors, such as self-efficacy, knowledge, social support, and access to environmental resources, leads to better exercise adherence. Longitudinal studies are also needed to clarify the temporal relationships between these factors and exercise adherence. These findings might inform future research and the development of multilevel approaches to support exercise adherence among stroke survivors across in-hospitalisation and post-discharge rehabilitation.

## Figures and Tables

**Table 1:** Summary of quality assessment using JBI critical appraisal checklist.

Author (Year)	JBI Critical Appraisal Checklist								Total (%)	Quality
	Q1	Q2	Q3	Q4	Q5	Q6	Q7	Q8		
Dong et al. (2025) [20]	✓	U	U	✓	U	✓	✗	✓	5.5 (68.75%)	Acceptable
Feng et al. (2025) [21]	✓	✓	✓	✓	U	✓	✓	✓	7.5 (93.75%)	Good
Long et al. (2025) [22]	✓	✓	✓	✓	U	✓	✓	✓	7.5 (93.75%)	Good
Xing et al. (2025) [23]	✓	U	✓	✗	U	✓	✓	✓	6 (75%)	Good
Yang et al. (2025) [24]	✓	✓	✓	✓	U	✓	✓	✓	7.5 (93.75%)	Good
Wo et al. (2024) [25]	✓	U	U	✓	U	✓	✓	✓	6.5 (81.25%)	Good
Wu et al. (2024) [26]	✓	✓	✓	✓	U	✓	✓	✓	7.5 (93.75%)	Good
Zhou et al. (2024) [27]	✓	✓	✓	✓	U	✓	✓	✓	7.5 (93.75%)	Good
Zhu et al. (2023) [28]	✓	✓	U	✓	U	✓	✓	✓	7 (87.50%)	Good
Chen et al. (2022) [29]	✓	U	U	✓	U	✓	✓	✓	6.5 (81.25%)	Good
Ping et al. (2022) [30]	✓	U	✓	✓	U	✓	✓	✓	7 (87.50%)	Good
Zhu et al. (2022) [31]	✓	✓	U	✓	U	✓	✓	✓	7 (87.50%)	Good
Fu et al. (2018) [32]	✓	U	U	✓	U	✓	✓	✓	6.5 (81.25%)	Good
Li et al. (2025) [33]	✓	✓	U	✓	U	✓	✓	✓	7 (87.50%)	Good
Zhang et al. (2025) [34]	✓	✓	✓	✓	U	✓	✓	✓	7.5 (93.75%)	Good
Lin et al. (2022) [35]	✓	U	✓	U	U	✓	✓	✓	6.5 (81.25%)	Good
Li et al. (2021) [36]	✓	U	U	✓	U	✓	✓	✓	6.5 (81.25%)	Good
Li et al. (2021) [37]	✓	✓	✗	✓	U	✓	✓	✓	6.5 (81.25%)	Good
Cao et al. (2020) [38]	✓	✓	U	✗	U	✓	✓	✓	6 (75%)	Good
Lian et al. (2019) [39]	✓	✓	✓	✓	U	✓	✓	✓	7.5 (93.75%)	Good
Zou et al. (2015) [40]	✓	✓	U	✓	U	✓	✓	✓	7 (87.50%)	Good
Zhang et al. (2015) [41]	✓	✓	✗	✓	U	✓	U	✓	6 (75%)	Good
Lin et al. (2013) [42]	✓	✓	U	✓	U	✓	✓	✓	7 (87.50%)	Good

✓ = Yes; ✗ = No; U = Unclear; Q1: Were the criteria for inclusion in the sample clearly defined; Q2: Were the study subjects and the setting described in detail; Q3: Was the exposure measured in a valid and reliable way; Q4: Were objective, standard criteria used for measurement of the condition; Q5: Were confounding factors identified; Q6: Were strategies to deal with confounding factors stated; Q7: Were the outcomes measured in a valid and reliable way; Q8: Was appropriate statistical analysis used.

**Table 2:** Characteristics of the included studies.

Author (Year)	Location	Sample Size	Age	Setting	Tool
Dong et al. (2025) [20]	Jiangsu	102	62.37 ± 8.12	In-hospital	CSFE
Feng et al. (2025) [21]	Hebei	304	≥60	In-hospital	EAQ
Long et al. (2025) [22]	Sichuan	307	69.41 ± 10.90	In-hospital	EAQ
Xing et al. (2025) [23]	Chongqing	227	18–59 (34.80%); 60–74 (41.90%); ≥75 (23.30%)	In-hospital	EAQ
Yang et al. (2025) [24]	Xinjiang	313	<45 (8.00%); 45–55 (22.00%); 56–65 (33.90%); >65 (36.10%)	In-hospital	EAQ
Wo et al. (2024) [25]	Jiangsu	149	≥60	In-hospital	EAQ
Wu et al. (2024) [26]	Shanxi	234	63.23 ± 11.64	In-hospital	EAQ
Zhou et al. (2024) [27]	Sichuan	421	≥60	In-hospital	EAQ
Zhu et al. (2023) [28]	Central, Eastern, and Northern China	397	50.64 ± 9.75	In-hospital	EAQ
Chen et al. (2022) [29]	Zhejiang	108	62.10 ± 13.30	In-hospital	EAQ
Ping et al. (2022) [30]	Anhui	62	18–60	In-hospital	EAQ
Zhu et al. (2022) [31]	Central, Eastern, and Northern China	412	18–64	In-hospital	EAQ
Fu et al. (2018) [32]	Chongqing	317	65.90 ± 10.20	In-hospital	EAQ
Li et al. (2025) [33]	Guangdong	201	64.20 ± 13.30	Post-discharge	EAQ
Zhang et al. (2025) [34]	Shandong	531	67.56 ± 10.27	Post-discharge	EAQ
Lin et al. (2022) [35]	Shanghai	534	67.64 ± 10.31	Post-discharge	EAQ
Li et al. (2021) [36]	Shandong	1250	55.39 ± 10.87	Post-discharge	EAQ
Li et al. (2021) [37]	Henan	217	<60 (42.40%); 60–75 (54.80%); >75 (2.80%)	Post-discharge	Self-developed
Cao et al. (2020) [38]	Henan	596	65.70 ± 10.76	Post-discharge	EAQ
Lian et al. (2019) [39]	Zhejiang	389	63.85 ± 13.61	Post-discharge	EAQ
Zou et al. (2015) [40]	Guangdong	120	67.41 ± 6.42	Post-discharge	EAQ
Zhang et al. (2015) [41]	Zhejiang	158	66.30 ± 5.60	Post-discharge	Self-developed
Lin et al. (2013) [42]	Henan	208	70.25 ± 9.08	Post-discharge	EAQ

Age is presented as reported by each study: mean ± SD, range, eligibility criterion, or age-group distribution; Tool refers to the measure used to assess exercise adherence; EAQ = Exercise Adherence Questionnaire; CSFE = Compliance scale of functional exercise; Self-developed = questionnaires developed by the original study authors.

**Table 3:** Exercise adherence outcomes of the included studies.

Author (Year)	Adherence Outcomes		
	Adherence Index	Adherence Level Distribution (%)	Overall Adherence Level
Dong et al. (2025) [20]	68.29%	N/A	Medium
Feng et al. (2025) [21]	65.88%	High: 24.00%; Medium: 66.80%; Low: 9.20%	Medium
Long et al. (2025) [22]	54.83%	N/A	Medium
Xing et al. (2025) [23]	68.6%	High: 41.00%; Medium: 41.80%; Low: 17.20%	Medium
Yang et al. (2025) [24]	72.96%	High: 50.79%; Medium: 44.41%; Low: 4.47%	Medium
Wo et al. (2024) [25]	62.18%	N/A	Medium
Wu et al. (2024) [26]	62.10%	N/A	Medium
Zhou et al. (2024) [27]	64.37%	N/A	Medium
Zhu et al. (2023) [28]	77.60%	N/A	High
Chen et al. (2022) [29]	61.20%	High: 14.80%; Medium: 63.90%; Low: 21.30%	Medium
Ping et al. (2022) [30]	70.73%	N/A	Medium
Zhu et al. (2022) [31]	77.71%	N/A	High
Fu et al. (2018) [32]	N/A	High: 10.73%; Medium: 21.77%; Low: 67.50%	Low
Li et al. (2025) [33]	65.21%	High: 33.80%; Medium: 45.80%; Low: 20.40%	Medium
Zhang et al. (2025) [34]	N/A	High: 42.60%; Medium: 32.80%; Low: 24.70%	Medium-to-high
Lin et al. (2022) [35]	N/A	High: 42.50%; Medium: 32.80%; Low: 24.70%	Medium-to-high
Li et al. (2021) [36]	53.89%	N/A	Medium
Li et al. (2021) [37]	57.85%	N/A	Medium
Cao et al. (2020) [38]	N/A	High: 17.80%; Medium: 56.90%; Low: 25.30%	Medium
Lian et al. (2019) [39]	66.19%	N/A	Medium
Zou et al. (2015) [40]	59.38%	N/A	Medium
Zhang et al. (2015) [41]	61.96%	N/A	Medium
Lin et al. (2013) [42]	61.98%	N/A	Medium

N/A indicates that the information was not reported in the study.

**Table 4:** Summary of factors associated with exercise adherence (In-hospital).

Variables		Positive Association, *n*	Negative Association, *n*	No Association, *n*	*n*/*N* (%)	Strength of Evidence
**Intrapersonal**	**Demographic factors**					
	Age		4		4/4; 100%	−−
	Gender (female)	1		1	1/2; 50%	?
	Marital status (married)	2		1	2/3; 67%	+
	Educational level	5		3	5/8; 63%	++
	Average income	2		2	2/4; 50%	?
	Employment status (employed)	1			1/1; 100%	NA
	Occupation (farmer)			1	0/1; 0%	NA
	Payment method (health insurance)	1		1	1/2; 50%	?
	**Health and clinical status factors**					
	Number of stroke episodes	1			1/1; 100%	NA
	Stroke severity		3		3/3; 100%	−−
	Time since stroke onset		1		1/1; 100%	NA
	Length of hospital stay		1		1/1; 100%	NA
	Presence of ICU stay		1		1/1; 100%	NA
	Frailty		1		1/1; 100%	NA
	Functional independence	5		1	5/6; 83%	++
	Muscle strength	1		1	1/2; 50%	?
	Perceived limb heaviness	1			1/1; 100%	NA
	Number of comorbidities			1	0/1; 0%	NA
	Diabetes	1			1/1; 100%	NA
	Hypertension			1	0/1; 0%	NA
	Hemiplegia		1		1/1; 100%	NA
	Communication impairment		1	1	1/2; 50%	?
	Insomnia			1	0/1; 0%	NA
	**Psychosocial and behavioural factors**					
	Knowledge	1			1/1; 100%	NA
	Hope	1			1/1; 100%	NA
	Subjective well-being	1			1/1; 100%	NA
	Self-efficacy	3			3/3; 100%	++
	Readiness for action	1			1/1; 100%	NA
	Exercise planning	1			1/1; 100%	NA
	Health beliefs	1			1/1; 100%	NA
	Psychological distress		2	1	2/3; 67%	−
	Personality		1		1/1; 100%	NA
	Illness perception		1		1/1; 100%	NA
	Alcohol consumption			1	0/1; 0%	NA
**Interpersonal**	Social support	3			3/3; 100%	++
	Family functioning	2			2/2; 100%	+
	Caregiver (paid helper)			1	0/1; 0%	NA
	Caregiver health status			1	0/1; 0%	NA
	Rehabilitation guidance/support			1	0/1; 0%	NA

Note. “*n*” indicates the number of studies reporting an association; “*n*/*N*” indicates the number and proportion of relevant studies reporting an association in the same direction out of the total studies; “+” indicates a positive association, “−” indicates a negative association, “0” indicates no association, and “?” indicates inconsistent findings; “++”, “−−” indicate sufficient consistency of evidence; NA indicates that further analysis was not possible.

## Data Availability

No new data were created or analyzed in this study. Data sharing is not applicable to this article.

## Supplementary Materials

The following supporting information can be downloaded at: https://www.mdpi.com/article/10.3390/healthcare14162475/s1, Supplementary File S1: Search strategies for Chinese and English databases.
