# Supplementary material for: Factors Associated with Exercise Adherence Across Settings Among Stroke Survivors in China: A Systematic Review of Cross-Sectional Studies
Source: Healthcare (Basel). 2026 Aug 10;14(16):2475. doi: 10.3390/healthcare14162475 (PMC13511845; doi:10.3390/healthcare14162475)
Supplement: Supplementary file 1 [file healthcare-14-02475-s001.zip › healthcare-4414389-supplementary.pdf]

Supplementary File S1

Note. Search strategies are reported using the original terms used in the searches. Chinese terms were used for Chinese databases, and English terms were used for English databases.

Search strategies for Chinese databases
Database 1: CNKI

Search	Query	Results
#1	SU=脑卒中 OR SU=中风 OR SU=卒中 OR SU=缺血性脑卒中 OR SU=脑梗 OR SU=脑血栓 OR SU=脑栓塞 OR SU=出血性脑卒中 OR SU=脑出血 OR SU=蛛网膜下腔出血 OR SU=脑血管意外	340,105
#2	SU=锻炼 OR SU=功能锻炼 OR SU=康复锻炼 OR SU=康复训练 OR SU=身体活动 OR SU=运动 OR SU=运动康复 OR SU=运动游戏 OR SU=运动疗法 OR SU=抗阻力运动 OR SU=抗阻训练 OR SU=力量训练 OR SU=力量练习 OR SU=有氧运动 OR SU=有氧训练 OR SU=耐力运动 OR SU=循环训练 OR SU=负重训练 OR SU=拉伸训练 OR SU=游泳 OR SU=步行 OR SU=自行车训练 OR SU=普拉提 OR SU=太极 OR SU=瑜伽	1,762,581
#3	SU=依从 OR SU=不依从 OR SU=依从性 OR SU=遵从 OR SU=配合	442,584
#4	SU=影响因素 OR SU=决定因素 OR SU=相关因素 OR SU=促进因素 OR SU=阻碍因素 OR SU=预测因素 OR SU=因素 OR SU=相关性	3,193,563
#5	#1 AND #2 AND #3 AND #4	196
#6	检索范围：学术期刊	101

Chinese Database 2: Wanfang

Search	Query	Results
#1	主题:(脑卒中 OR 中风 OR 卒中 OR 缺血性脑卒中 OR 脑梗 OR 脑血栓 OR 脑栓塞 OR 出血性脑卒中 OR 脑出血 OR 蛛网膜下腔出血 OR 脑血管意外)	621,241
#2	主题:(锻炼 OR 功能锻炼 OR 康复锻炼 OR 康复训练 OR 身体活动 OR 运动 OR 运动康复 OR 运动游戏 OR 运动疗法 OR 抗阻力运动 OR 抗阻训练 OR 力量训练 OR 力量练习 OR 有氧运动 OR 有氧训练 OR 耐力运动 OR 循环训练 OR 负重训练 OR 拉伸训练 OR 游泳 OR 步行 OR 自行车训练 OR 普拉提 OR 太极 OR 瑜伽)	2,611,649
#3	主题:(依从 OR 不依从 OR 依从性 OR 遵从 OR 配合)	1,083,790
#4	主题:(影响因素 OR 决定因素 OR 相关因素 OR 促进因素 OR 阻碍因素 OR 预测因素 OR 因素 OR 相关性)	7,347,642
#5	#1 AND #2 AND #3 AND #4	641
#6	检索范围：期刊论文	332

Chinese Database 3: Weipu

Search	Query	Results
#1	M=(脑卒中 OR 中风 OR 卒中 OR 缺血性脑卒中 OR 脑梗 OR 脑血栓 OR 脑栓塞 OR 出血性脑卒中 OR 脑出血 OR 蛛网膜下腔出血 OR 脑血管意外)	355,482
#2	M=(锻炼 OR 功能锻炼 OR 康复锻炼 OR 康复训练 OR 身体活动 OR 运动 OR 运动康复 OR 运动游戏 OR 运动疗法 OR 抗阻力运动 OR 抗阻训练 OR 力量训练 OR 力量练习 OR 有氧运动 OR 有氧训练 OR 耐力运动 OR 循环训练 OR 负重训练 OR 拉伸训练 OR 游泳 OR 步行 OR 自行车训练 OR 普拉提 OR 太极 OR 瑜伽)	848,321
#3	M=(依从 OR 不依从 OR 依从性 OR 遵从 OR 配合)	229,339
#4	M=(影响因素 OR 决定因素 OR 相关因素 OR 促进因素 OR 阻碍因素 OR 预测因素 OR 因素 OR 相关性)	1,302,913
#5	#1 AND #2 AND #3 AND #4	66

Supplementary File S1

Search strategies for English databases
Database 1: PubMed

Search	Query	Results
#1	"stroke"[MeSH Terms] OR "cerebrovascular disorders"[MeSH Terms] OR "brain ischemia"[MeSH Terms] OR "intracranial hemorrhages"[MeSH Terms] OR "intracranial embolism and thrombosis"[MeSH Terms] OR "brain infarction"[MeSH Terms]	467,867
#2	"stroke*"[Title/Abstract] OR "poststroke*"[Title/Abstract] OR "post stroke*"[Title/Abstract] OR "post-stroke*"[Title/Abstract] OR "cerebral stroke*"[Title/Abstract] OR "cerebrovascular accident*"[Title/Abstract] OR "cerebral vascular accident*"[Title/Abstract] OR "cerebrovascular infarct*"[Title/Abstract] OR "cerebral infract*"[Title/Abstract] OR "cerebral ischemi*"[Title/Abstract] OR "cerebral ischaemi*"[Title/Abstract] OR "cerebral thrombo*"[Title/Abstract] OR "cerebral emboli*"[Title/Abstract] OR "brain ischemi*"[Title/Abstract] OR "brain ischaemi*"[Title/Abstract] OR "brain infarct*"[Title/Abstract] OR "ischaemic stroke*"[Title/Abstract] OR "ischemic stroke*"[Title/Abstract] OR "cerebellar stroke*"[Title/Abstract] OR "cerebellar infarct*"[Title/Abstract] OR "cerebellar ischaemi*"[Title/Abstract] OR "cerebellar ischemi*"[Title/Abstract] OR "cerebellar thrombo*"[Title/Abstract] OR "cerebellar emboli*"[Title/Abstract] OR "vertebrobasilar stroke*"[Title/Abstract] OR "vertebrobasilar infarct*"[Title/Abstract] OR "vertebrobasilar ischemi*"[Title/Abstract] OR "vertebrobasilar ischaemi*"[Title/Abstract] OR "vertebrobasilar emboli*"[Title/Abstract] OR "vertebrobasilar thrombo*"[Title/Abstract] OR "basal ganglia stroke*"[Title/Abstract] OR "basal ganglia ischaemi*"[Title/Abstract] OR "basal ganglia ischemi*"[Title/Abstract] OR "basal ganglia infarct*"[Title/Abstract] OR "thrombotic stroke*"[Title/Abstract] OR "embolic stroke*"[Title/Abstract] OR "haemorrhagic	477,959

	stroke*"[Title/Abstract] OR "hemorrhagic stroke*"[Title/Abstract] OR "brain haemorrhag*"[Title/Abstract] OR "brain hemorrhag*"[Title/Abstract] OR "cerebral haemorrhag*"[Title/Abstract] OR "cerebral hemorrhag*"[Title/Abstract] OR "cerebellar haemorrhag*"[Title/Abstract] OR "cerebellar hemorrhage*"[Title/Abstract] OR "intracerebral haemorrhag*"[Title/Abstract] OR "intracerebral hemorrhag*"[Title/Abstract] OR "intracranial haemorrhag*"[Title/Abstract] OR "intracranial hemorrhag*"[Title/Abstract] OR "subarachnoid haemorrhag*"[Title/Abstract] OR "subarachnoid hemorrhag*"[Title/Abstract] OR "basal ganglia haemorrhag*"[Title/Abstract] OR "basal ganglia hemorrhag*"[Title/Abstract]	
#3	#1 OR #2	684,490
#4	"exercise"[MeSH Terms] OR "exercise therapy"[MeSH Terms] OR "stroke rehabilitation"[MeSH Terms] OR "rehabilitation"[MeSH Terms]	625,481
#5	"exercise therap*"[Title/Abstract] OR "kinesiotherap*"[Title/Abstract] OR "physical therap*"[Title/Abstract] OR "physiotherap*"[Title/Abstract] OR "rehabilitat*"[Title/Abstract] OR "exercis*"[Title/Abstract] OR "functional exercise"[Title/Abstract] OR "physical activit*"[Title/Abstract] OR "physical training"[Title/Abstract] OR "resistance training"[Title/Abstract] OR "strength training"[Title/Abstract] OR "aerobic exercise"[Title/Abstract] OR "endurance training"[Title/Abstract] OR "circuit training"[Title/Abstract] OR "weight training"[Title/Abstract] OR "weight bearing"[Title/Abstract] OR "ergomet*"[Title/Abstract] OR "treadmill"[Title/Abstract] OR "running"[Title/Abstract] OR "jogging"[Title/Abstract] OR "walking"[Title/Abstract] OR "stair climbing"[Title/Abstract] OR "stair-climbing"[Title/Abstract] OR	1,154,298

	"cycling"[Title/Abstract] OR "rowing"[Title/Abstract] OR "pilates"[Title/Abstract] OR "tai chi"[Title/Abstract] OR "taiji"[Title/Abstract] OR "yoga"[Title/Abstract] OR "stretching"[Title/Abstract] OR "swimming"[Title/Abstract] OR "exergam*"[Title/Abstract]	
#6	#4 OR #5	1,423,962
#7	"patient compliance"[MeSH Terms] OR "treatment adherence and compliance"[MeSH Terms]	363,632
#8	"adhere*"[Title/Abstract] OR "nonadhere*"[Title/Abstract] OR "complian*"[Title/Abstract] OR "noncomplian*"[Title/Abstract] OR "cooperat*"[Title/Abstract]	664,183
#9	#7 OR #8	957,931
#10	"determinant*"[Title/Abstract] OR "correlate*"[Title/Abstract] OR "facilitat*"[Title/Abstract] OR "barrier*"[Title/Abstract] OR "predict*"[Title/Abstract] OR "factor*"[Title/Abstract] OR "associat*"[Title/Abstract]	12,585,553
#11	"china"[Affiliation]	3,645,072
#12	"clinical trial"[Filter] OR "randomized controlled trial"[Filter] OR "review"[Filter] OR "systematic review"[Filter] OR "meta-analysis"[Filter]	4,915,231
#13	#3 AND #6 AND #9 AND #10 AND #11 NOT #12	131

Database 2: Web of Science

**Search**	**Search terms**	**Results**
#1	TS=("stroke*" OR "poststroke*" OR "post stroke*" OR "post-stroke*" OR "cerebral stroke*" OR "cerebrovascular accident*" OR "cerebral vascular accident*" OR "cerebrovascular infarct*" OR "cerebral infract*" OR "cerebral ischemi*" OR "cerebral ischaemi*" OR "cerebral thrombo*" OR "cerebral emboli*" OR "brain ischemi*" OR "brain ischaemi*" OR "brain infarct*" OR "ischaemic stroke*" OR "ischemic stroke*" OR "cerebellar stroke*" OR "cerebellar infarct*" OR "cerebellar ischaemi*" OR "cerebellar ischemi*" OR "cerebellar thrombo*" OR "cerebellar emboli*" OR "vertebrobasilar stroke*" OR "vertebrobasilar infarct*" OR "vertebrobasilar ischemi*" OR "vertebrobasilar ischaemi*" OR "vertebrobasilar emboli*" OR "vertebrobasilar thrombo*" OR "basal ganglia stroke*" OR "basal ganglia ischaemi*" OR "basal ganglia ischemi*" OR "basal ganglia infarct*" OR "thrombotic stroke*" OR "embolic stroke*" OR "haemorrhagic stroke*" OR "hemorrhagic stroke*" OR "brain haemorrhag*" OR "brain hemorrhag*" OR "cerebral haemorrhag*" OR "cerebral hemorrhag*" OR "cerebellar haemorrhag*" OR "cerebellar hemorrhage*" OR "intracerebral haemorrhag*" OR "intracerebral hemorrhag*" OR "intracranial haemorrhag*" OR "intracranial hemorrhag*" OR "subarachnoid haemorrhag*" OR "subarachnoid hemorrhag*" OR "basal ganglia haemorrhag*" OR "basal ganglia hemorrhag*")	635,578
#2	TS=("exercise therap*" OR "kinesiotherap*" OR "physical therap*" OR "physiotherap*" OR "rehabilitat*" OR "exercis*" OR "functional exercise" OR "physical activit*" OR "physical training" OR "resistance training" OR "strength training" OR "aerobic exercise" OR "endurance training" OR "circuit training" OR "weight training" OR "weight bearing" OR "ergomet*" OR "treadmill" OR "running" OR "jogging" OR "walking" OR "stair climbing" OR "stair-climbing" OR "cycling" OR "rowing" OR "pilates" OR "tai	2,004,243

	chi" OR "taiji" OR "yoga" OR "stretching" OR "swimming" OR "exergam*")	
#3	TS=("adhere*" OR "nonadhere*" OR "complian*" OR "noncomplian*" OR "cooperat*")	1,224,997
#4	TS=("determinant*" OR "correlate*" OR "facilitat*" OR "barrier*" OR "predict*" OR "factor*" OR "associat*")	21,259,545
#5	CU=("china")	10,390,788
#6	DT=("review" OR "meta-analysis" OR "clinical Trial" OR "randomized controlled trial")	3,073,867
#7	#1 AND #2 AND #3 AND #4 AND #5 NOT #6	179
